# Effect of Polymers on Behavior of Ultra-High-Strength Concrete

**DOI:** 10.3390/polym14132585

**Published:** 2022-06-25

**Authors:** Ola A. Mayhoub, Aref A. Abadel, Yousef R. Alharbi, Moncef L. Nehdi, Afonso R. G. de Azevedo, Mohamed Kohail

**Affiliations:** 1 Department of Civil Engineering, El Gazera High Institute for Engineering and Technology, Cairo 11571, Egypt; eng_olamayhoub@yahoo.com; 2 Department of Civil Engineering, College of Engineering, King Saud University, Riyadh 11421, Saudi Arabia; yrharbi@ksu.edu.sa; 3 Department of Civil Engineering, McMaster University, Hamilton, ON L8S 4L8, Canada; 4 LECIV—Civil Engineering Laboratory, UENF—State University of the Northern Rio de Janeiro, Av. Alberto Lamego, 2000, Campos dos Goytacazes, Rio de Janeiro 28013-602, RJ, Brazil; afonso@uenf.br; 5 Structural Engineering Department, Faculty of Engineering, Ain Shams University, Cairo 11535, Egypt; m.kohail@eng.asu.edu.eg

**Keywords:** styrene butadiene rubber, steam curing, steel fibers, tensile strength

## Abstract

The development of ultra-high-performance concrete (UHPC) is still practically limited due to the scarcity of robust mixture designs and sustainable sources of local constituent materials. This study investigates the engineering characteristics of Styrene Butadiene Rubber (SBR) polymeric fiber-reinforced UHPC with partial substitution of cement at 0, 5 and 20 wt.% with latex polymer under steam and air curing techniques. The compressive and tensile strengths along with capillary water absorption and sulfate resistance were measured to evaluate the mechanical and durability properties. Scanning Electron Microscopy (SEM) was carried out to explore the microstructure development and hydration products in the designed mixtures under different curing regimes. The results indicated that the mixtures incorporating 20 wt.% SBR polymer achieved superior compressive strength at later ages. Additionally, the tensile strength of the polymeric UHPC without steel fibers and with 20% polymers was enhanced by 50%, which promotes the development of novel UHPC mixtures in which steel fibers could be partially replaced by polymer, while enhancing the tensile properties.

## 1. Introduction

Concrete is the world’s most-consumed manufactured material. Substantial research has been carried out to enhance the engineering properties, rheology, durability, and sustainability of various types of concrete. Ultra-high-performance concrete (UHPC) has emerged as an innovative structural composite with superior engineering properties. Due to exclusion of coarse aggregates, the UHPC matrix typically has a homogenous microstructure with high particle-packing density [1,2,3,4,5]. Furthermore, incorporating microfibers into the UHPC matrix significantly enhances its flexural strength, ductility, and toughness [6,7,8]. Fiber inclusion can restrain the coalescence, growth, and propagation of microcracks and thus, mitigate brittle fractures [9,10,11]. Additionally, fibers increase the toughness energy owing to the crack bridging effect [12,13,14,15]. Nevertheless, uniform dispersion of the fibers throughout the cementitious matrix is of particularly great significance [6,16,17]. The congregation and nonuniform distribution of fibers in the matrix can compromise the consolidation, mechanical strength, and ductility of UHPC [18,19].

To enhance the flexural strength and brittleness of ultra-high-strength cementitious matrices, organic polymers can be integrated into UHPC mixtures [20,21]. Various organic polymers, including ethylene-vinyl acetate, poly-acrylamide, methyl-cellulose, styrene acrylates, and styrene-butadiene latex, can prompt such beneficial effects [22,23,24,25,26]. Owing to their superior strength, eco-efficiency, and good workability, polymer-modified cement-based composites have been broadly utilized in the construction of civil infrastructures along with the repair and rehabilitation of concrete structures [27,28,29]. Styrene-butadiene rubber latex, epoxy resin, and poly-acrylamide are among the polymers typically used in cementitious composites in the form of aqueous solutions or dispersible powders [30,31,32]. More recently, butyl benzene latex, carboxylic styrene-butadiene latex, neoprene matrix, styrene-acrylic matrix (SAE), and polyacrylate latex have been utilized in latex-cement mortars [20]. Butyl benzene latex modified cement mortars have indicated exceptional toughness while requiring less water when compared to different types of polymers. Thus, they have been prevalently used as adhesives in repairing materials for civil engineering structures [33,34,35]. The polymer latex may be categorized into two main groups: (i) SBR latex with only enhanced physical properties; (ii) carboxylic styrene-butadiene latex with enhanced physical and chemical properties.

Polymers coat the cement particles and fill in the pores within the cement hydration products. This physical densification reduces the porosity of the cementitious matrix and thereby, enhances the mechanical properties. Furthermore, lower porosity can reduce the intrusion of hostile substances, such as chloride and sulfate ions, and bridge microcracks to enhance ductility and toughness [34,35,36,37]. The inclusion of polymers also favorably affects the flowability and workability of conventional concrete as water-reducing admixtures have been widely used [35,38,39].

The alkaline nature of most of the polymers helps them to maintain their material properties in the alkaline cementitious environment. Polymers with long chains are created by the polymerization of different numbers of monomers. Styrene butadiene rubber (SBR) is a homopolymer or copolymer of 1,3–butadiene and styrene as monomers. So in this study, SBR has been used to investigate its behavior in ultra-high-strength fiber-reinforced concrete.

Despite the pertaining research on the engineering properties of polymer-modified cement-based composites, few studies have investigated the influence of polymers on the behavior of ultra-high-strength fiber-reinforced concrete under different curing regimes [40,41]. To address this research gap, the current study evaluates the engineering properties of air- and steam-cured high-strength fiber-reinforced concrete mixtures incorporating SBR latex polymer at two different dosages. Compressive strength, tensile strength, sorptivity, and scanning electron microscopy (SEM) were used to investigate the strength and microstructure development of the mixtures cured under different curing regimes.

## 2. Experimental Program

### 2.1. Materials

Ordinary Portland cement (OPC) type I in accordance with ASTM C150, non-densified grey silica fume (SF) with the mean particle size of 0.1 µm in compliance with ASTM C 1240, and ground granulated blast furnace slag (GGBFS) were utilized as binders [42,43]. The chemical and the physical properties of the used binders are shown in Table 1.

To enhance the particle packing density, silica sand with a grain size ranging from 150 to 600 µm was used as the fine aggregates. Additionally, quartz powder with a particle size ranging from 10 to 45 µm was utilized as the micro filler. An aqueous solution of poly-carboxylate superplasticizer types G and F that complied with ASTM C494 was utilized to improve workability. Styrene Butadiene Rubber latex was incorporated at 0, 5, and 20 wt.% as a replacement for cement. Steel fibers with a length of 30 mm and a diameter of 0.75 mm were used at 2 v.%.

### 2.2. Mixing and Curing Conditions

Eight various UHPC mixtures were designed to evaluate the mechanical and durability properties. The mixing process was started with mixing dry ingredients for one minute. Thereafter, the mixture of water and superplasticizer along with SBR polymers were added to the mixer. The entire mixture was mixed for 4 min as the mixer speed was gradually increased until the desired consistency was attained. A vibrating table was utilized to adequately compact the UHPC mixture after casting into molds. The mixtures were cured using two different curing regimes: air and steam curing. In steam curing, samples were steam cured at 90 °C for seven days and then cured in 20 °C water until the age of 28 days.

### 2.3. Mix Design Proportions

The designation and mix proportion of the UHPC mixtures are outlined in Table 2. The water-to-binder ratio of 0.16 was used for all mixtures. The total amount of cementitious materials was 1549 kg/m^3^. SBR polymers were added as a replacement for cement at the dosage of 5 and 20 wt.%. In mixture designations, character P refers to the incorporated polymer, 5% and 20% indicate the replacement level of cement with polymer, character F refers to the incorporated steel fibers, and characters S and A demonstrate the steam and air curing regimes respectively.

### 2.4. Test Procedure

#### 2.4.1. Mechanical Strength

The compressive strength of 100 mm cube specimens was measured at 7, 28 and 90 days of curing. Triplicate samples were tested for each age. A compressive test machine (ADR Touch SOLO 2000 BS EN Compression Machine) with a capacity of 2000 kN and a loading rate of 14 MPa per minute was used. The machine was manufactured in the United Kingdom. The speed of the mixer of 12 rpm and the mixing was at ambient room temperature of 24 °C. The splitting tensile strength was tested on 100 × 200 mm cylindrical specimens at the age of 28 days according to ASTM standard ASTM C496 [44].

#### 2.4.2. Sorptivity Test

Cylindrical specimens having a height of 50 mm and a diameter of 100 mm were utilized to perform the sorptivity test as per ASTM C1585-04 standard [45]. The sorptivity test was performed on specimens at 90 days to evaluate the capacity of UHPC specimens to absorb and transport water via capillary pressure as a measure of durability.

#### 2.4.3. Scanning Electron Microscopy (SEM) Analysis

SEM was used to examine the effects of SBR and steel fiber inclusion along with the steam curing on the microstructure development of the UHPC samples. The SEM samples were collected from fractured specimens after the compressive strength test at 90 days. SEM samples were coated with gold for better resolution. The SEM device was VEGA3 TESCAN series. It is a family of modern, fully PC-controlled scanning electron microscopes with a tungsten-heated filament.

#### 2.4.4. Sulfate Resistance

100-mm cubic specimens, which were steam cured for seven days followed by moist curing until 28 days were fully immersed in a concentration by mass of 10% Na_2_SO_4_ solution for 60 days to determine the resistance of the UHPC mixtures against sulfates at later ages. The cubic specimens were then tested in the compressive test machine to investigate the impact of sulfates exposure on the compressive strength of the UHPC.

## 3. Results and Discussion

### 3.1. Mechanical Strength

**Figure 1:**
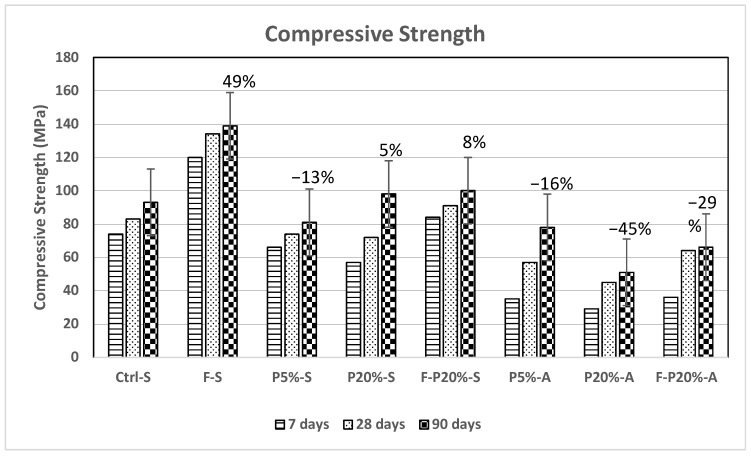
Compressive strength of mixtures at 7, 28 and 90 days.

Figure 1 compares the compressive strength of the mixtures tested at 7, 28 and 90 days of curing. It was observed that a noticeable enhancement in the compressive strength of the steam cured mixtures was achieved compared to those cured in air. Therefore, steam curing favorably affected the strength development of polymeric UHPC at early and later ages. This observation complies with the studies in the literature which reported the enhancement of compressive strength in polymeric concrete [38]. What is interesting in the compressive strength measurements is that incorporating polymers into UHPC prompts insignificant improvement in the compressive strength after 7 and 28 days. Nevertheless, a considerable increase in the compressive strength of the mixture containing 20 wt.% polymer (i.e., P20%-S) at 90 days was achieved by 5–8% enhancement. This may be related to the continued physical activities and chemical reactions of the polymer latex at later ages. Chemical reactions between polymers and cement can activate cement reaction through chemical bonding connections. The results have shown that the SBR react with Ca (OH)^2^ to form a new network structure. This may be attributed to the polymer particles that covered the surface of cement gel particles. As the water reacts with the cement in the hydration process, the polymer fills in the capillary pores and hence the polymer particles form a polymer sealing layer and create bridging bonds. This would enhance the physical structure of the cementitious matrix, therefore improving the microstructure of the polymer-cement system. Hence it is concluded that the addition of polymers enhances the long-term compressive strength. Interestingly, although 7- and 28-day compressive strength of mixtures incorporating steel fibers are higher than those without steel fibers, the 90-day compressive strength of mixtures with and without steel fibers are comparably improved with 49% enhancement as shown in Figure 1.

**Figure 2:**
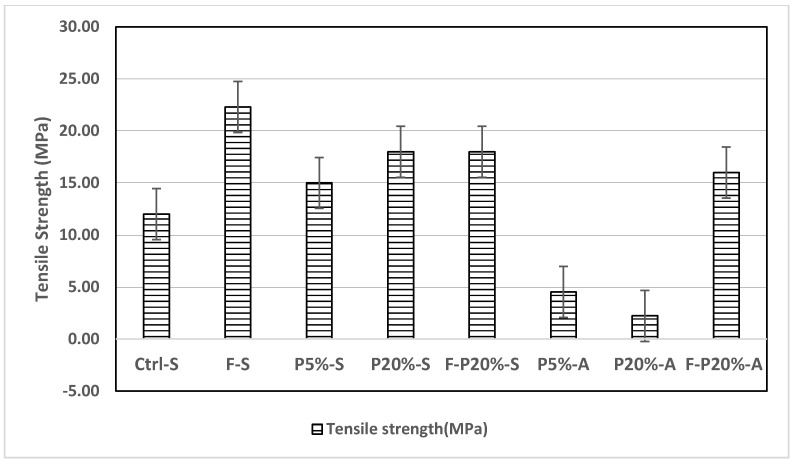
Tensile of mixtures at 28 days.

Figure 2 displays the split tensile strength of the mixtures tested at 28 days. Mixtures incorporating steel fibers exhibited the highest tensile strength which is in good agreement with previous studies in the literature [39]. It should be noted that the tensile strength of polymeric UHPC with 5 wt.% polymer and no steel fiber inclusion was increased by 25%. Additionally, when 20 wt.% polymer was utilized, the tensile strength improved by 50% compared to the control sample. This demonstrates that using polymers can significantly enhance the tensile performance of UHPC mixtures without using steel fibers. It is well known that fibers have major disadvantages such as high corrosion risk, negative impact on the structural surface finishing, and increase in the economic expenses [46,47]. Therefore, replacing steel fibers with polymers may mitigate such disadvantages.

### 3.2. SEM Analysis

**Figure 3:**
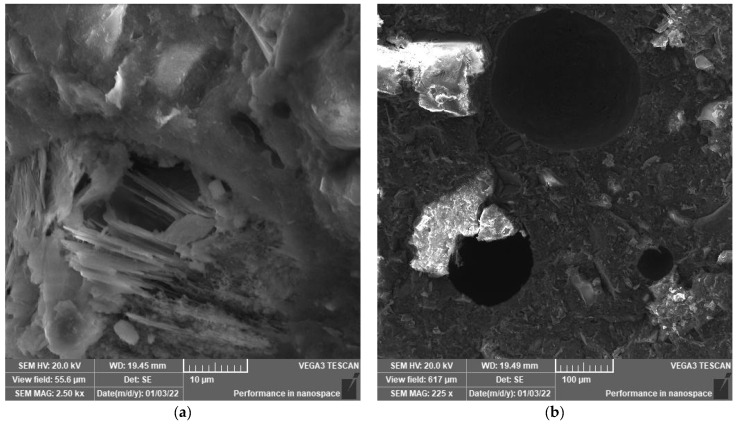
(**a**) SEM at 2.5 k× magnification and (**b**) SEM at 225× magnification. SEM images of mix Ctrl-S.

**Figure 4:**
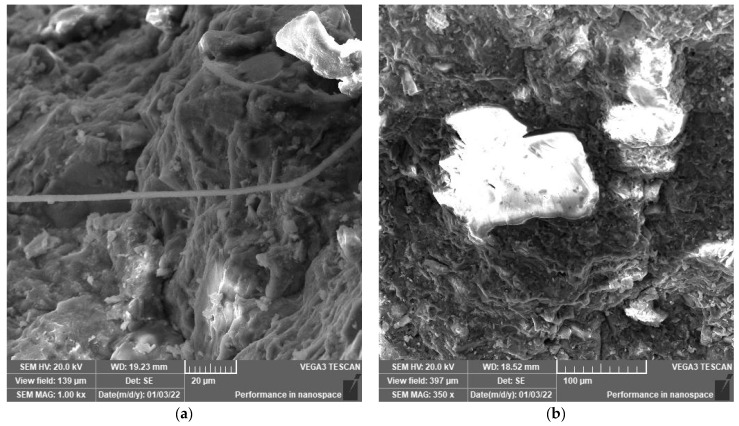
(**a**) SEM at 1.0 k× magnification and (**b**) SEM at 350× magnification. SEM images for mix F-S.

**Figure 5:**
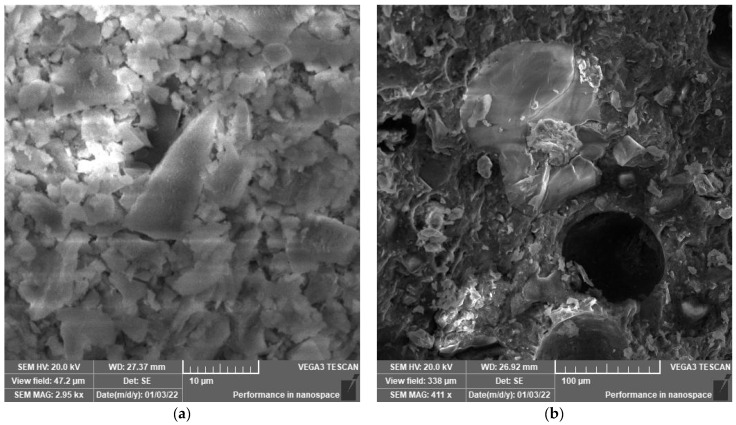
(**a**) SEM at 2.95 k× magnification and (**b**) SEM at 411× magnification. SEM images for mix P5%-S.

**Figure 6:**
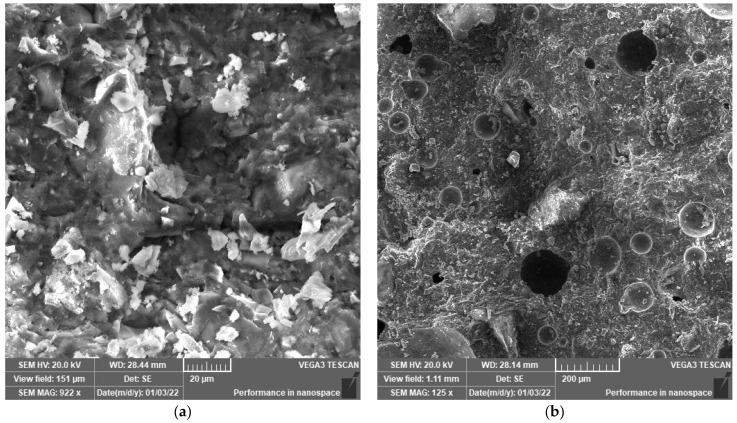
(**a**) SEM at 922× magnification and (**b**) SEM at 125× magnification. SEM images for mix P20%-S.

**Figure 7:**
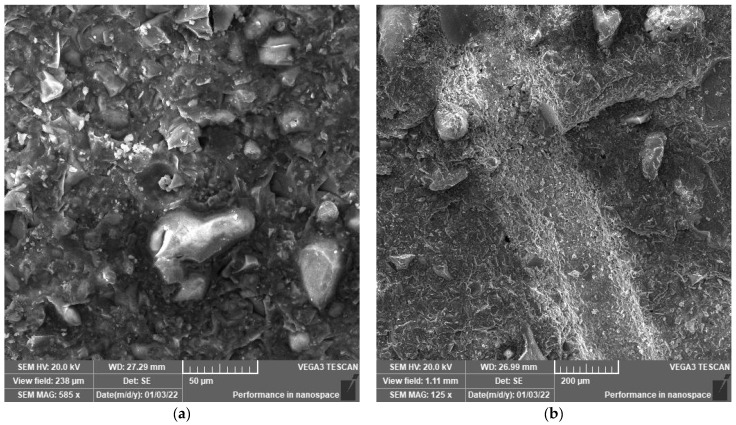
(**a**) SEM at 585× magnification and (**b**) SEM at 125× magnification SEM images for mix F-P20%-S.

**Figure 8:**
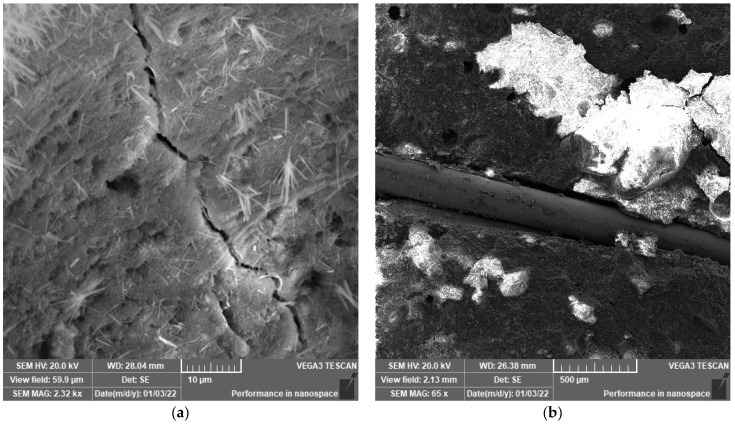
(**a**) SEM image of mix P5%-A at 2.32kx magnification and (**b**) SEM image of mix F-P20%-A at 65× magnification.

SEM analysis was performed to explore the microstructure development of polymeric UHPC mixtures at the age of 90 days. The reason to apply this age is to assure that the cement has gained nearly the maximum hydration reaction. SEM images shown in Figure 3, Figure 4, Figure 5, Figure 6, Figure 7 and Figure 8 were captured from the failure surface of the samples crushed in compressive strength tests. For all mixtures investigated herein, a high compacted gel-like matrix can be observed, which is calcium silicate hydrate (CSH). Additionally, the brighter parts in the matrix are anhydrate cement grains and the darker parts surrounding the anhydrate particles are the C-S-H gel. Figure 3 illustrates the SEM images of the control mix.

Figure 4 shows the link between the steel fibers and the surrounding cement matrix. It is observed that the addition of fibers had a good impact on bridging the crack. Furthermore, the orientation of fibers on the fracture plane favorably affects the mechanical behavior, which is in great agreement with the results obtained from compressive and tensile strength tests.

Figure 5 and Figure 6 indicate the SEM images of the mixtures incorporating polymers. It is clear that replacing cement with polymer resulted in the presence of more anhydrate cement particles. In particular, the hydration products are considerably lower in the case of mixtures with 20 wt.% polymer inclusions. This indicates the dilution effect due to polymer addition which can restrain the hydration reaction of cement and thus, decrease the formation of CSH gel at early ages. Thus, the microstructure of the developed polymeric UHPC mixtures with high polymer content was not dense enough at early ages. However, at later ages, the microstructure development in such mixtures continued such that a dense microstructure and high mechanical properties were achieved. The reason to perform SEM test at later ages (i.e., 90 days) is to assure that the cement has gained nearly the maximum hydration reaction.

SEM analysis demonstrated that there is an enhancement in the bond strength between the polymer and the surrounding matrix. This result complies with the studies in the literature which reported the improved bond strength between the polymer and cement matrix, and therefore enhancement in the mechanical strengths [20,35,39]. Ultimately, it can be concluded that utilizing polymers may bring about compacted microstructure along with superior mechanical properties in UHPC mixtures. Previous studies exhibited that latex possesses a compact microstructure along with a less porous transition zone and non-connected voids [39]. The SEM images captured from various locations in this study also revealed no significant cracks in mixtures incorporating polymers, indicating the desirable effect of polymers on bridging microcracks in the cement matrix.

Pervious investigations reported enhancement in the interfacial zone in the mixtures which could be observed through a decrease in the quantity of calcium hydroxide and pores attributed to the presence of the polymer in the cementitious matrix. The decrease in the calcium hydroxide can be revealed through the reactions of the carboxylic group of the copolymer particles with Ca^2+^ ions released during the cement hydration process. This finding agrees with several past studies [48].

Another interesting finding is that the pore size distribution was finer in steam cured pastes due to the increased degree of binder activation. Therefore, polymeric UHPC has favorable mechanical and durability properties due to the microstructure development and constituent composition. The kind and concentration of polymers and cement influence their hydration [20]. This is evident comparing the mixes incorporating 5 wt.% polymer with those with 20% polymer replacement.

Figure 7 illustrate that the polymer was uniformly distributed in the matrix. Polymer interacts in the cement matrix and hardens on the surface of hydrating cement particles. As a result, the polymer generates a network action in the cement mortar when the water content in the matrix decreases. This signifies the importance of reducing water content in the mixes incorporating polymer and the limited strength gain in mixes containing polymer.

Such findings are in great agreement with the research studies in the literature. Accordingly, the polymer action may be divided into three phases:

when the polymer is added to a thoroughly mixed cement mortar, the polymer particles in the matrix are homogeneously dispersed.

Polymer is moderately enclosed in the capillary pores as the number of available water drops. Polymer particles form a covering polymeric layer when they agglomerate on the hydration gel surface. The active groups in polymer molecules can create unique bridge connections with cement hydration products, strengthening the physical structure of the hardened concrete and therefore, increasing the density of the polymeric cement matrix.

This in turn might fill the bigger gaps in the matrix. As the hydration process continues, the polymer matrix hydrolysate interacts with Ca(OH)_2_ to develop a new network matrix. These crosslinked network structure materials connect both hydrated and anhydrate particles, boosting the structural morphology of the concrete matrix [20].

Figure 8 shows that the microstructure of air-cured concrete contains a higher number of pores compared to the steam cured specimens in which the microstructure was fully packed with a homogeneous structure. Additionally, air curing caused larger crack widths in the matrix. In contrast, the steam cured samples exhibited cracks with considerably smaller widths. The concrete matrix tended to be more compact in the moisture-cured specimen, and the width of cracks in the interfacial transition zone (ITZ) significantly decreased. This is owing to the high temperature, which promotes the hydration of the cement and the chemical reaction of the polymers. The SEM images comply with the assumptions in this study and assist in understanding the impact of different curing regimes along with the effect of polymer inclusion in UHPC mixtures.

### 3.3. Capillary Water Absorption

**Figure 9:**
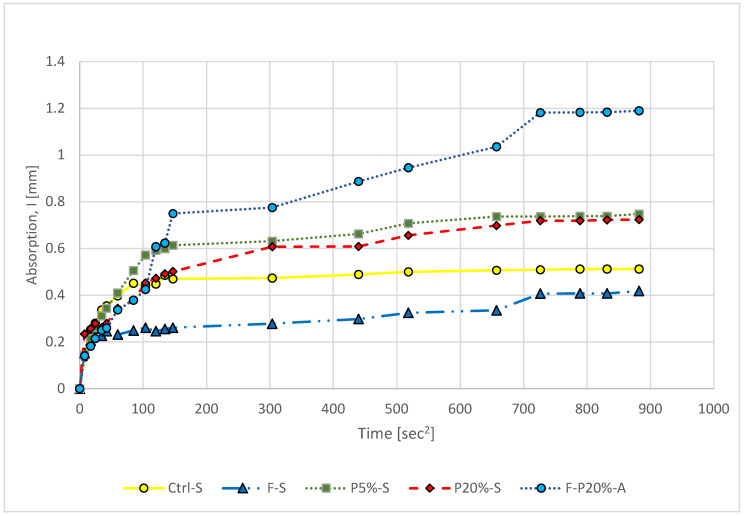
Capillary water absorption of polymeric UHPC mixtures.

Figure 9 displays the capillary water absorption curves of mixtures studied herein. The reduction in sorptivity indicates that the mixture has a very dense microstructure, which is generally one of the most critical variables influencing the mechanical characteristics and concrete durability. This may be related to the importance of replacing the water content in the mix design with added polymers due to the increase of the water content in the polymer ingredient. The results of the compressive strength tests revealed that the method of steam curing considerably improved the strength development of high-strength concrete. This was also observed in the results of sorptivity measurement of mixture F-P20%-A which indicated the highest water absorption due to air curing. The absorption level was reduced with the incorporation of 20% polymers thus, polymers can be utilized in steel corrosion cessation. The addition of polymers may cause excess water content which may be the reason for the presence of more pores and hence increase the water absorption. It is obvious from Figure 9 that the water absorption rate of Ctrl-S and F-S mixtures are lower than that of polymer-containing mixes.

### 3.4. Sulfate Resistance

**Figure 10:**
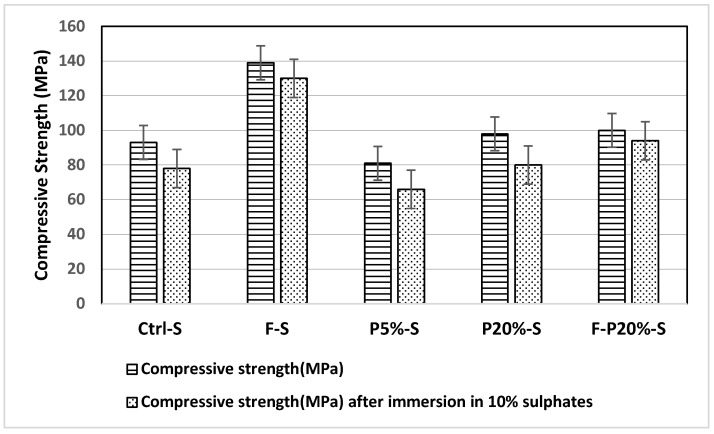
Sulfate resistance of mixtures after immersion in 10% sodium sulfate solution.

The sulfate resistance of UHPC mixtures was evaluated after they were fully immersed in a 10% Na_2_SO_4_ solution for 60 days. After 60 days, the concrete specimens were removed from the solutions for inspection, and the compressive strength test was carried out to determine the deterioration of concrete under sulfate attacks. Figure 10 illustrates the compressive strength of mixtures before and after immersion in the sodium sulfate solution. Accordingly, the concrete surface was damaged, and its strength was significantly reduced. This damage was accompanied by the gradual intrusion of external sulfate ions into concrete and hence both physical and chemical corrosion reactions occurred. This observation can be noticed from the result of the compressive strength test after the immersion in the sulfate solution. It was noted that the concrete compressive strength decreased approximately by 18.5% in both mixtures incorporating 5 and 20 wt.% polymer. Furthermore, in specimens with steel fiber and 20 wt.% polymer inclusions, the reduction in compressive strength did not exceed 6%. Nevertheless, in the control mix, the compressive strength was reduced by 33%. This demonstrates the enhancement in the sulfate resistance of UHPC after the addition of both polymers and steel fibers. This complies with the studies in the literature that reported the increased sulfate resistance of concrete after polymer incorporation [20].

## 4. Conclusions

The mechanical properties, durability, and microstructure of SBR polymer UHPC were investigated. The following conclusions can be drawn:

- The microstructure of the UHPC incorporating high polymer content was dense and high compressive strength at later ages was achieved. This is attributed to the polymer matrix that enclosed cement particles and in turn prevented cement particles’ contact with water, resulting in a delay in the hydration of anhydrate cement particles and microstructural development.
- The inclusion of polymers can substantially enhance the tensile strength of UHPC. The tensile strength of polymeric UHPC without steel fibers and with 20 wt.% polymer was enhanced by 50%. This demonstrates the possibility of replacing steel fibers with polymers to enhance tensile strength.
- Steam curing was the most appropriate curing condition for polymeric UHPC.
- The effectiveness of polymers varied widely depending on the percentage of polymers and the curing techniques.
- It was concluded that using polymer may increase the water absorption in concrete. This may be related to the importance of replacing the water content in the mix design with added polymers due to the increase of the water content in the polymer ingredients.
- An enhancement was observed in the sulfate resistance of UHPC after the addition of both polymers and steel fibers.
- Further studies are needed to investigate the effect of other curing techniques along with the optimum polymer content.

## Figures and Tables

**Table 1:** Chemical composition and physical properties of OPC, SF and GGBFS.

Component/Property	OPC	SF	GGBFS
SiO_2_	20.8	91.5	39.8
Al_2_O_3_	4.82	0.47	11.2
Fe_2_O_3_	4	1.53	1.2
MgO	1.4	1.6	7.6
CaO	62.6	0.89	34.4
Na_2_O	0.4	0.22	0.2
SO_3_	2.54	0.43	0.46
K_2_O	0.24	1.11	-
TiO_2_	-	-	-
Loss on ignition	1.9	2.3	1.2
Specific Gravity	3.17	2.20	2.85
Specific Surface area (m^2^/g)	0.350	20	15

**Table 2:** Mixture design of UHPC mixtures [kg/m^3^].

Component (kg/m^3^)	CEM 1	Silica Fume	Slag	Silica Sand	Quartz Powder	Steel Fiber	Polymer	HRWR *	Water	Curing Regime
Ctrl-S	1126	282	141	422	141	-	-	23	245	Steam
F-S	1126	282	141	422	141	156	-	23	245	Steam
P5%-S	1070	282	141	422	141	-	56.3	23	245	Steam
P20%-S	901	282	141	422	141	-	225	23	245	Steam
F-P20%-S	901	282	141	422	141	156	225	23	245	Steam
P5%-A	1070	282	141	422	141	-	56.3	23	245	Air
P20%-A	901	282	141	422	141	-	225	23	245	Air
F-P20%-A	901	282	141	422	141	156	225	23	245	Air

* HRWR = high range water reducer or superplasticizer.

## Data Availability

The data presented in this study are available on request from the corresponding author.
